# Assessing calcification effects in TEVAR procedures: a computational analysis

**DOI:** 10.1007/s10237-025-01998-9

**Published:** 2025-09-12

**Authors:** Giulia De Campo, Anna Ramella, Sara Barati, Giulia Luraghi, Virginia Fregona, Maurizio Domanin, Robin Heijmen, Santi Trimarchi, Francesco Migliavacca

**Affiliations:** 1 Department of Chemistry, Materials and Chemical Engineering ‘Giulio Natta’, Politecnico Di Milano, Milan, Italy; 2 AllStent S.R.L, Milan, Italy; 3 Department of Clinical Sciences and Community Health, University of Milan, Milan, Italy; 4 Fondazione IRCCS Ca’ Granda Ospedale Maggiore Policlinico, Milan, Italy; 5 Department of Cardiothoracic Surgery, Radboud University Medical Centre, Nijmegen, The Netherlands

**Keywords:** Stent graft, Finite element analysis (FEA), Thoracic endovascular aortic repair, In silico medicine

## Abstract

Thoracic endovascular aortic repair (TEVAR) procedure is sometimes discouraged from clinical guidelines in the presence of calcifications and thrombus along the sealing zones. This computational study aims to understand which is the effect of calcification on stent graft displacement after TEVAR procedure, simulated in a patient-specific anatomy with a penetrating aortic ulcer (PAU).

A patient-specific anatomy without calcification is taken as reference, and four models with idealized calcifications positioned in different regions and with different material properties are analyzed. Opening area, von Mises stresses and contact pressures are evaluated to provide a reliable comparison between the calcified (Ca) and the non-calcified models (noCa), and among the calcified models themselves.

Comparing qualitatively the Ca and noCa models, no particular changes in the stent graft apposition are observed. In addition, in the Ca models the opening area results lower with respect to the noCa models, but no significant differences are observed among the Ca models. Regarding the von Mises stresses, it seems that the calcifications act as load-bearing structures, absorbing the stresses and reducing them on the aorta. Decreasing the Young modulus of the calcifications, this effect is reduced. Higher contact pressures are observed when the highest Young’s modulus of calcification is adopted, with all Ca models having greater pressures than the noCa model.

From this analysis, the stent graft seems to be positioned correctly inside the aorta, even in the presence of calcifications. In this setting, the calcifications seem to reduce the stresses on the aorta, thus reducing the likelihood of aneurysm rupture.

**Supplementary Information:**

The online version contains supplementary material available at 10.1007/s10237-025-01998-9.

## Introduction

Thoracic endovascular aortic repair (TEVAR) was approved by FDA in 2005. From then, it has become the most common therapy for treatment of thoracic aneurysm (Findeiss and Cody 2011; Nation and Wang 2015). TEVAR is a minimally invasive technique which consists of placing of a stent graft in the pathological tract to replace the blood fluid dynamics.

In the latest years, computational models are gaining importance thanks to their ability of developing high-fidelity models to reproduce cardiovascular procedures and be used as a predictive tool in the preclinical decision-making process (Van Bogerijen et al. 2013; Ramella et al. 2022). They developed a high-fidelity model to predict the stent graft placement and deployment through a FEA simulation in accordance with the Validation & Verification (V&V) protocol by the American Society of Mechanical Engineering (ASME) (Aldieri et al. 2023).

When conducting this simulation, it is crucial to consider not only the patient’s anatomy (arch types I, arch type II, arch type III) and pathology (e.g., thoracic aortic aneurysm (TAA), penetrating aortic ulcer (PAU)), but also the presence of calcifications and thrombi. These factors might significantly impact the device apposition, potentially resulting in endoleaks or stent migration, two of the most common complications of the TEVAR procedure (Upchurch et al. 2021).

TEVAR guidelines discourage the procedure when highly calcified aortas are present, in particular in the landing zone regions, due to the possibility of developing endoleak (Upchurch et al. 2021; Grabenwöger et al., 2012); nevertheless, clinicians often proceed with the operation off-label despite these recommendations because of clinical necessities (Le Bars et al. 2022).

In the thoracic aorta, calcifications are predominantly found in the aortic arch (70% of the cases), followed by the descending aorta and the ascending aorta (Gui et al. 2024). When calcifications are present, a stiffening, thickening and a reduced compliance of the aortic wall are observed (Gui et al. 2024). Previous studies included calcifications in in silico models, evaluating the von Mises stress and the peak wall stress (PWS) on the aorta, but without simulating the stent graft implantation. They analyzed the effect of the calcification when the blood pressure is applied to the aortic wall. Meier et al. (2010) and Marra et al. (2006) analyzed the stresses in models with and without calcification when the blood pressure goes from 80 to 120 mmHg, including the prestress condition on the aorta, while Speelman et al. (2007) analyzed only the effect of the blood flow, without including the aorta prestress. Only McLennan et al. (2022) decided to assess the impact of calcification during endovascular aortic repair (EVAR) procedure; however, the clinical procedure itself was not explained in the paper (Gui et al. 2024; O’Leary et al. 2015).

Calcifications in these studies were modeled using two main approaches: explicitly, considering aorta and calcifications as separate parts, or implicitly, by assigning the material properties of the calcifications in the correspondent part of the aorta (McLennan et al. 2022). Furthermore, calcifications were modeled with hyperelastic, homogeneous, incompressible and isotropic material following the Mooney–Rivlin formulation (Li et al. 2008; Huang et al. 2001; McLennan et al. 2022; O’Leary et al. 2015), as linear elastic materials, using neo-Hookean model (Speelman et al. 2007; Maier et al. 2010), or with linear elastic materials (Luraghi et al. 2019). For what concerns the mechanical properties, discrepancies have been found in the literature, with Young modulus that ranged from 2.75 MPa (Speelman et al. 2007; Loree et al. 1994) to 20 GPa (Marra et al. 2006). Those values resulted from different mechanical testing of calcification and aortic wall.

Loore et al. in 1994 (Loree et al. 1994), after testing mechanically the calcifications embedded in the aortic tissue, found an elastic modulus that ranged from 0.182 to 2.75 MPa. Li et al. (2008) and Speelman et al. (2007), performing numerical simulations using the experimental values of Loore, adopting an explicit and implicit model, respectively, found an increase in the PWS (peak wall stress) of the aorta of 14% and 22%, respectively. Later on, Marra et al. in 2006 (Marra et al. 2006) measured through nanoindentation the calcification mechanical properties, finding a Young modulus of 20 GPa. However, no computational studies using these data were found. Lastly, Maier et al. in 2010 (Maier et al. 2010) performed other experimental testing on high calcified tissues and found a Young modulus of 50 MPa. They also performed a computational analysis, adopting an explicit model. They analyzed the PWS and the von Mises stresses on the aorta, finding different results from the one stated previously. They observed that the calcifications seemed to have a load-bearing behavior, and they did not find an increase in PWS on the aorta.

In this context, the primary aim of this study is to analyze, computationally, the effects of calcifications on a patient-specific anatomy suffering from penetrating aortic ulcer (PAU) after a TEVAR procedure, testing different calcification geometries and mechanical properties, to help understand how the different models can affect the outcome of the clinical procedure. The second aim is to explore the role of calcifications in influencing stent graft apposition and assess their potential contribution to clinical complications.

## Material and method

### Clinical data

A patient-specific anatomy has been used for this study. The patient presented an arch type III, with a PAU in correspondence of the arch and without calcifications. The aorta measured 24 mm in the landing zone and clinicians decided to perform TEVAR with a 28 × 28x100 proximal free-flow Valiant Captivia® stent graft (Medtronic, Inc., MN, USA). A postoperative computed tomography (CT) scan confirmed the correct positioning of the endograft. Pre- and post-op CT scan were segmented using VMTK (Orobix s.r.l.) software. The patient provided informed consent for the treatment and the use of the clinical data.

### Numerical model

#### Stent graft and aorta models

**Fig. 1:**
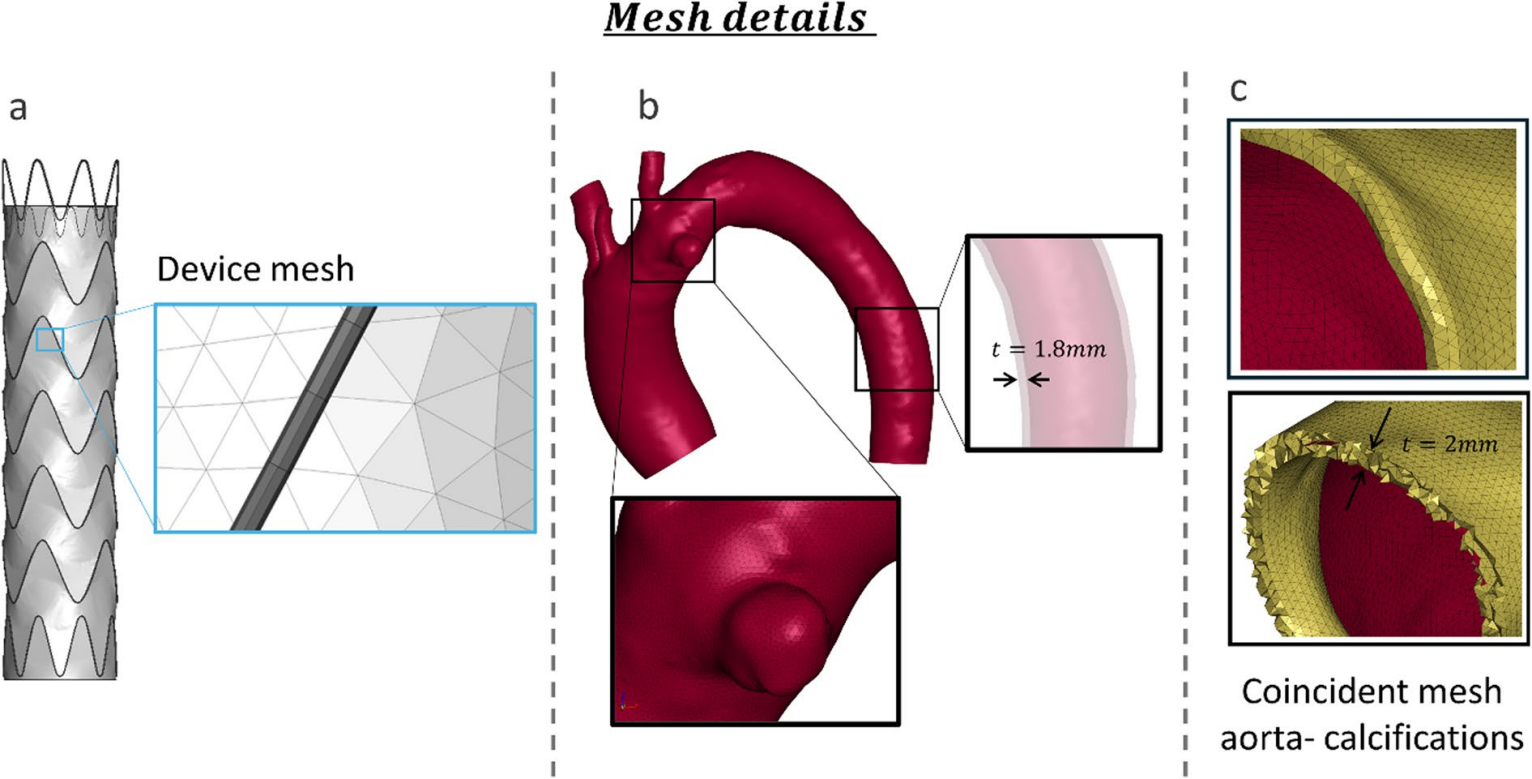
**a** Detailed mesh of the stent graft with coincident mesh between stent and graft. **b** Detailed mesh of the aorta, including a zoomed-in view of the pathology and thickness (t). **c** Detailed mesh of the calcifications, highlighting the thickness (t)

The 28 × 28 × 100 Valiant Captivia (Medtronic, Inc., MN, USA) stent graft was modeled following Ramella et al. 2022 (Ramella et al. 2022). The stent was discretized with linear beam elements with an average size of 0.5 mm with a section of 0.5 mm. The graft was discretized with triangular membrane elements with an average size of 0.5 mm and a thickness of 0.1 mm (Fig. 1a). Nitinol shape memory material formulation for the stent and a fabric material formulation for the graft was adopted. Material parameters followed the one presented by Ramella et al. (2022) from a calibration and validation analysis (Ramella et al. 2022). These parameters are reported in the online Appendix for a better comprehension. The patient-specific aorta lumen was segmented semiautomatically from preoperative CT images using the software VMTK (Orobix s.r.l.). This process generated an STL model representing the patient-specific anatomy. The resulting geometry was then smoothed to obtain a uniform model and subsequently meshed using ANSA (Beta CAE). The aorta was discretized with bi-linear triangular shell elements with an average size of 0.5 mm and a constant thickness of 1.8 mm. The mesh size was chosen to be consistent with that of the device to facilitate contact between the aorta and the device (Fig. 1b). The aorta material was modeled with a linear elastic material (Ramella et al. 2023a) (online Appendix). Prestress of the aorta was included in the model using the inverse elastostatic method implemented in ANSYS Mechanical (Ansys Inc., Canonsburg, PA, USA) (Ramella et al. 2024). The aorta was then re-pressurized by applying a pressure of 80 mmHg using LS-Dyna software (Ansys Inc., Canonsburg, PA, USA), since the CT was acquired during the diastolic phase.

The TEVAR simulation followed the “tracking method” developed by Ramella et al. 2022. Briefly, the device, which was in a prestressed state was crimped and displaced inside the aorta along the vessel centerline until the proximal landing zone was reached and then the device was gradually deployed (Ramella et al. 2022). Soft-penalty-based contacts were defined between the stent graft and aorta and calcifications. Supplementary details can be found in the online Appendix.

#### Calcification models

**Fig. 2:**
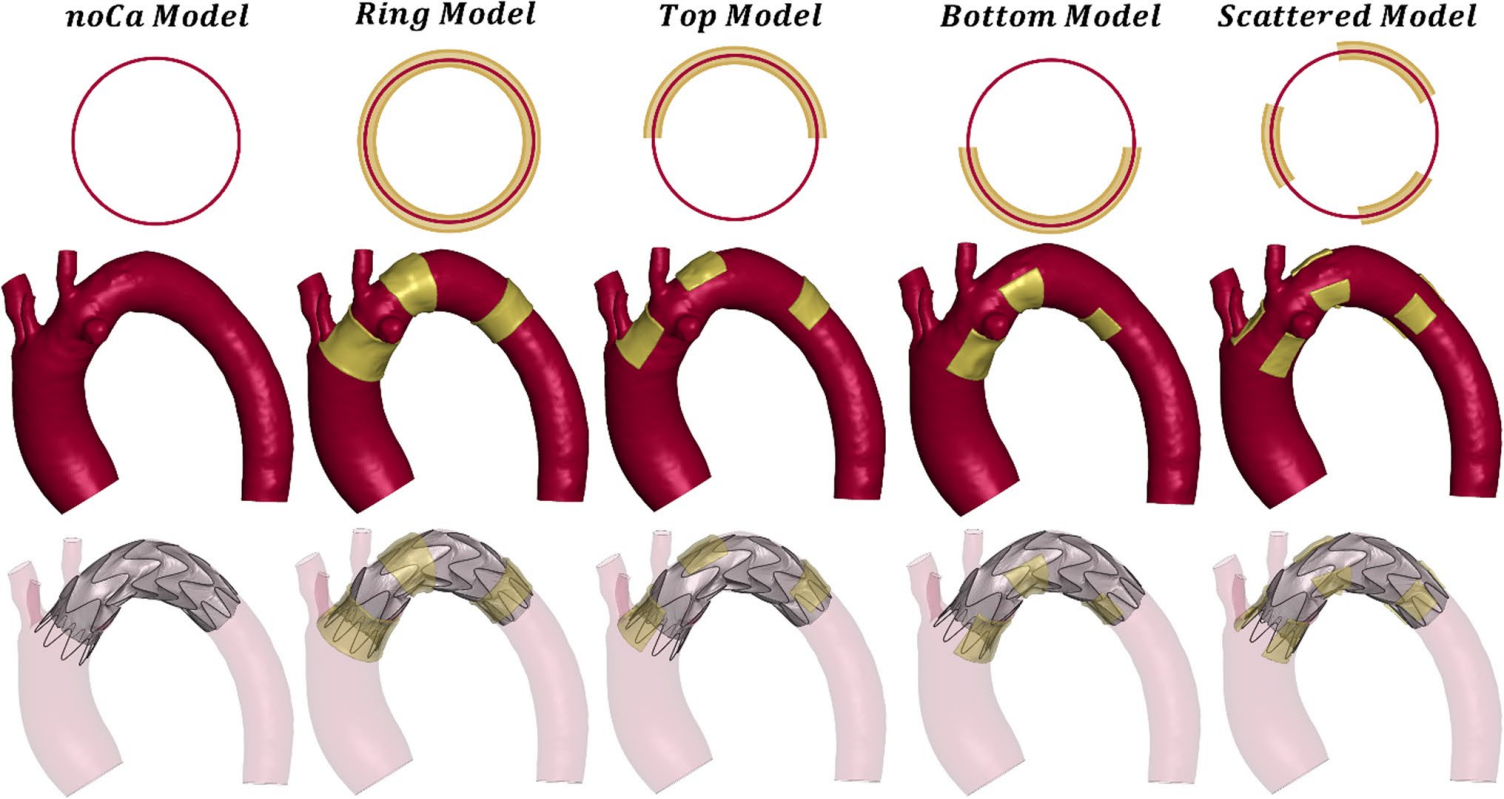
Model illustration and qualitative comparison of the outcome of the simulation in the five models

The calcifications were modeled with linear tetrahedral elements with an element size of 0.5 mm to match the aorta and to correctly capture the calcification geometry. The mesh is coincident with the aorta in both the internal and external parts (Fig. 1c). The aorta layer indeed crossed the calcification which grows in the internal part of the lumen and in the intima layer of the aorta with a total thickness of around 2 mm. According to Vos et al., who analyzed 40 patients, most calcifications exceeded a thickness of 1.5 mm (Vos et al. 2023). In contrast, severe calcifications are typically classified as having a thickness greater than 4 mm (Desai et al. 2018). For this study, we selected a thickness within this range and we maintained it consistently across all models. With the aim of analyzing the impact of calcification on TEVAR procedure, four different idealized calcification models are proposed in this study (Fig. 2).

The *ring model* (RM) was characterized by an extensive plaque which completely surround the proximal and distal stent graft landing zone and the arch. In the *top* and *bottom models* (TM and BM), the calcification occupied half of the space covered by the RM, concentrated in the bottom and in the upper part of the aorta. Eventually the *scattered model* (SM), whose calcified areas occupy one-third of the RM, reproduced a more common representation of the calcification, where calcium is spotted on the aorta, without following a pattern (Wong et al. 2012). Nevertheless, RM, TM and BM can be associated to a high calcified aorta, such as porcelain aortas or coral reef aortas (Le Bars et al. 2022, Desai et al. 2018, Ladich et al., 2016).

The degree of severity of the calcification was calculated with the Aortic Calcification Index (ACI) (Takayama et al. 2016). The aorta was divided into slices from the proximal to the distal part. Each slice was divided into 12 pieces, and depending on how many pieces have the calcification, the ACI was calculated with the following equation:

$${\text{ACI}} \left( \% \right) = \frac{s}{12 \left( n \right) } \times 100$$

where *s* is the total score of calcifications in all the slices and *n* is the number of planes that cut the aorta (Takayama et al. 2016). Based on the ACI quantification, the RM exhibited the highest degree of calcification, with an ACI of 50%. The SM followed with an ACI of 36%, while the TM and BM had indices of 30% and 28%, respectively.

No prestress was applied on calcifications. Pathologically, they grow over the years; therefore, we chose to apply blood pressure to the calcifications only after the aorta was re-pressurized. To clarify, the prestress and its subsequent re-pressurization to include the residual stresses was performed exclusively on the aorta. After that, a constant pressure of 80 mmHg was applied to the entire model, including both the aorta and the calcifications. As for the aorta, linear elastic material was adopted for calcification. Furthermore, three different elastic moduli of the calcification were tested: 2.75 MPa, 50 MPa and 20 GPa (online Appendix). Those values were taken from the literature (Loree et al. 1994, Reeps et al., 2013, Marra et al. 2006).

#### Assessment of the calcifications on TEVAR outcome

A total of thirteen simulations of TEVAR procedure were performed on the different geometric distribution of the calcifications and material properties, as described above. These simulations included the four calcified models each tested with three different Young modulus. Additionally, a reference model without calcification was included for a comparative analysis. On the outcome of these simulations, qualitative and quantitative parameters were analyzed.

Qualitative comparison regarded the stent overlapping with the CT-post for the reference model (without calcification, real patient) and the stent struts comparison among the four models with calcifications, while the quantitative analysis was performed on the evaluation of the error in the validation process, which consist in the opening area measurements of the simulation and segmentation models (Ramella et al. 2022), the calculation of the opening areas differences among all the models and on the assessment of von Mises stresses and contact pressures.

von Mises stresses and contact pressure values were extracted from the simulation results. The stresses were evaluated and averaged across the entire aorta. For what concern the contact pressure, the 10 maximum values were extracted and mediated. Lastly, the locations of the maximum values were represented on the aorta geometry to understand where the maximum contact pressure points were located.

## Results

### Validation with patient-specific post-TEVAR CT scan

**Fig. 3:**
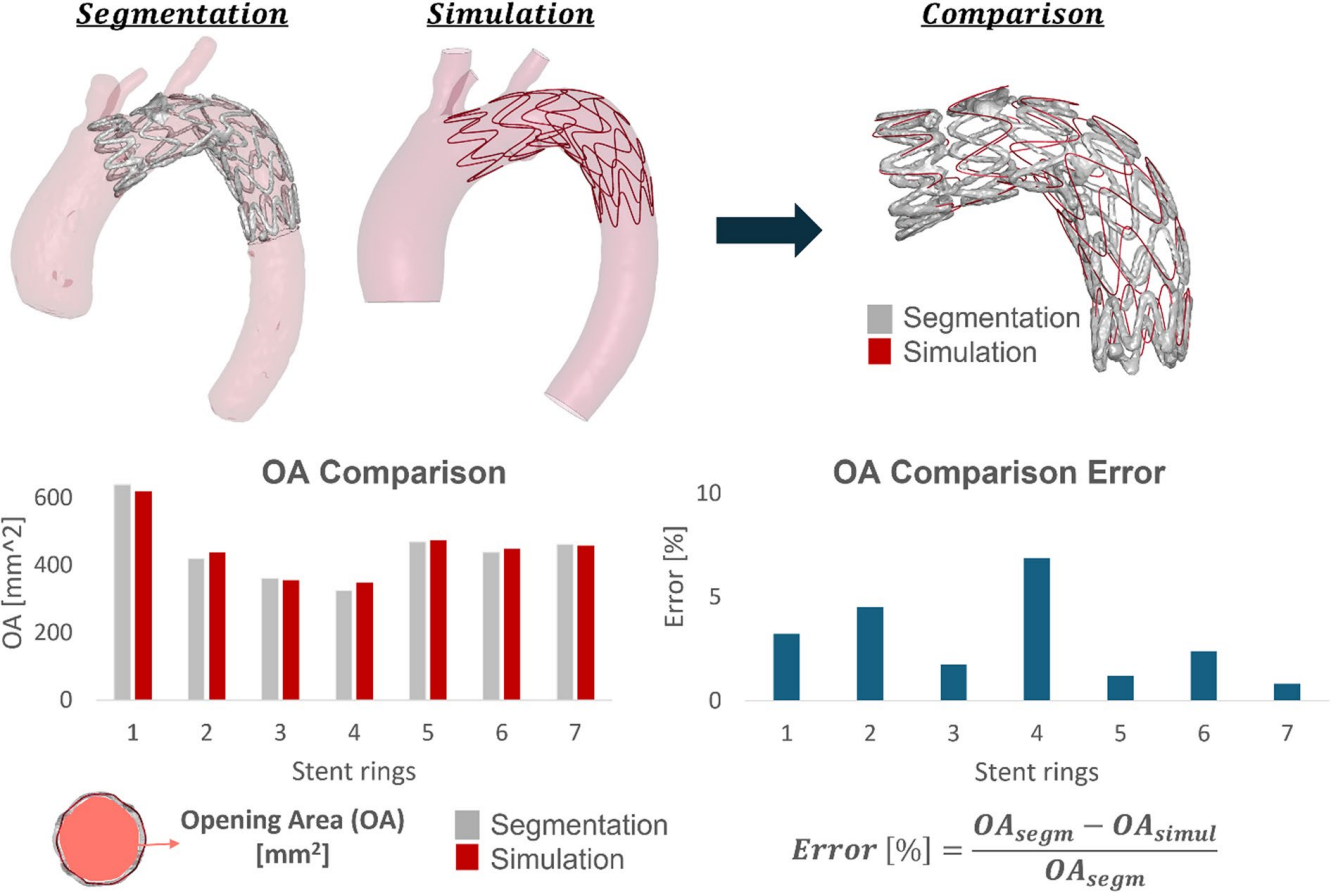
Validation process: comparison of opening area (OA) of segmented and simulated models

In Fig. 3, both the qualitative and quantitative comparison between the simulation and the segmentation model can be observed. The simulation showed good agreement with the CT-post, with errors in the opening area lower than 7% for all the stent struts.

### Impact on the stent graft deployment

In this section, RM, TM, BM and SM were referred to as Ca models, while the reference patient-specific simulation was named as noCa model. Comparing the models from a qualitative point of view, the stent graft apposition looked the same for all the models, suggesting a good stent graft apposition even in the presence of calcifications (Fig. 2). The qualitative comparison was made with the Ca model with Young modulus of 20 GPa, as it was the worst scenario, being the calcified region of four orders of magnitude stiffened than the aortic wall.

**Fig. 4:**
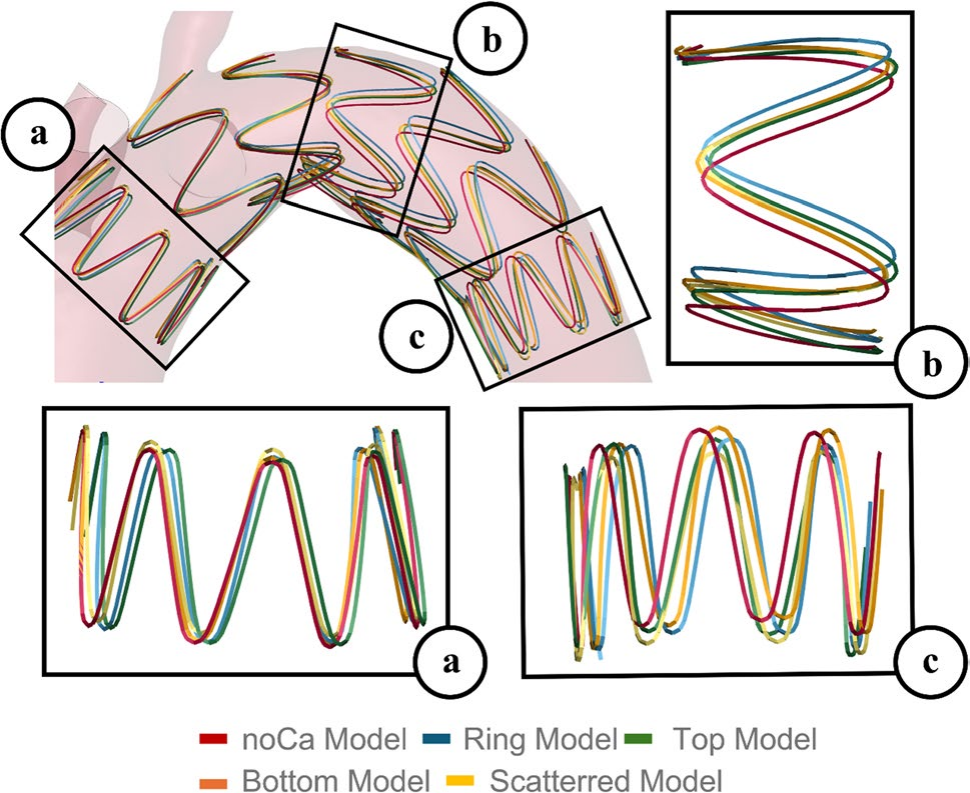
Qualitative comparison of the stent struts, with a particular attention on the first, fourth and seventh ring

Subsequently, a qualitative comparison of the stent struts in contact with the calcifications was also made. The first three struts demonstrated better alignment between the Ca and noCa models, whereas the fourth and final struts showed lower alignment (maximum rotation of calcium/no calcium of 8.72°, which shows more than 200% increase with respect to the first three rings), likely due to strut rotation. This suggested that the calcification in the arch may have caused a rotation of the device during the tracking phase. In Fig. 4, the first, fourth and seventh rings are highlighted to illustrate the behavior described above.

**Fig. 5:**
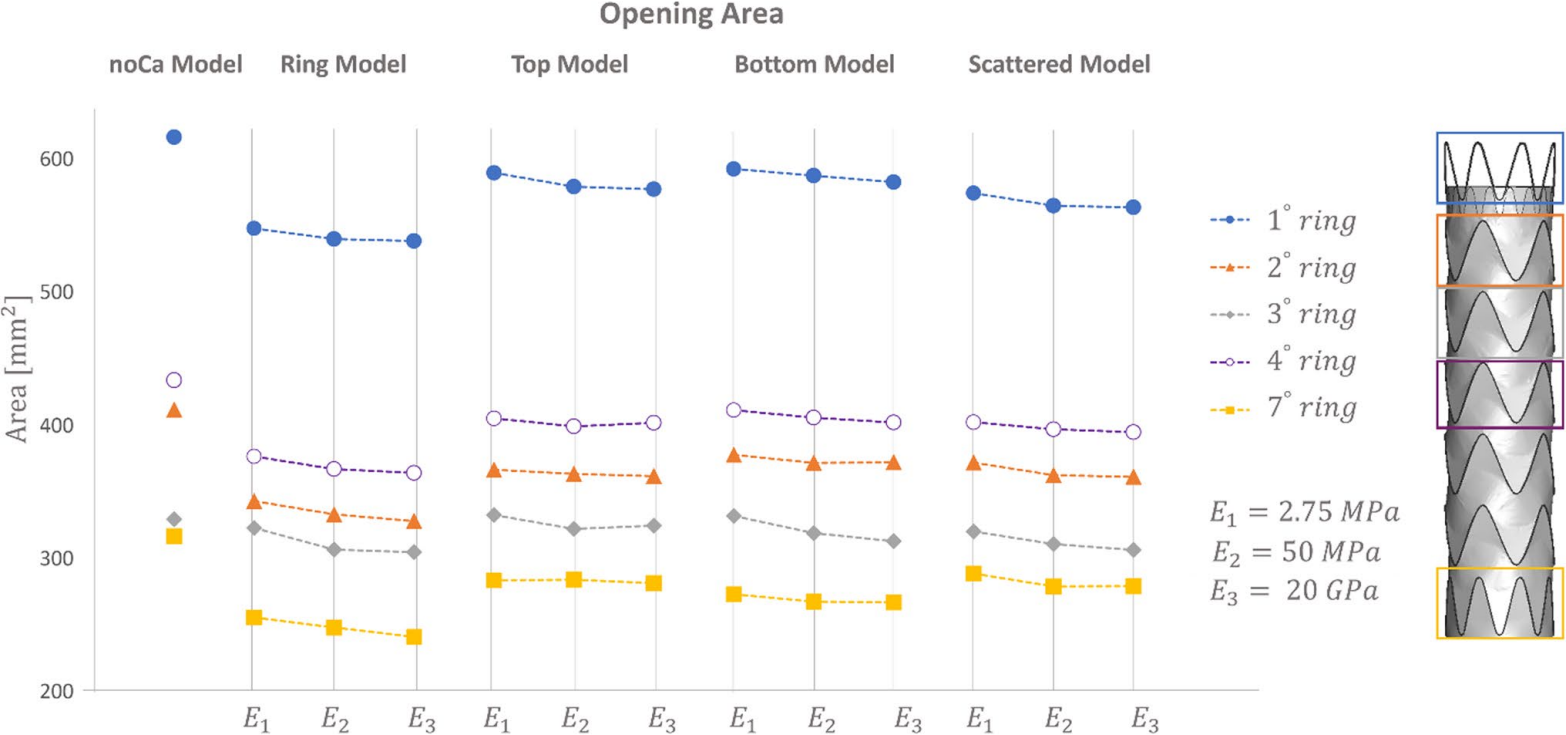
Quantification of the opening area for the Ca and noCa models for each considered E

The opening area of the noCa model was compared with the ones of the Ca models, considering only the stent struts in contact with the calcification, which were the ones highlighted in Fig. 4. The opening area resulted higher in all the struts of the noCa model respect to the Ca models. The highest discrepancies were reached with the RM, where the biggest decrease in the opening area was experienced, in particular for the second and fourth ring comparison. On the other hand, the third ring presented opening area values in the Ca models closer to the noCa models. TM, BM and SM showed lower decreases in the opening area as compared to the noCa model. To conclude, increasing the stiffness of the calcifications for all the Ca models, the opening area was slightly reduced (Fig. 5).

### Impact on the von Mises stresses

**Fig. 6:**
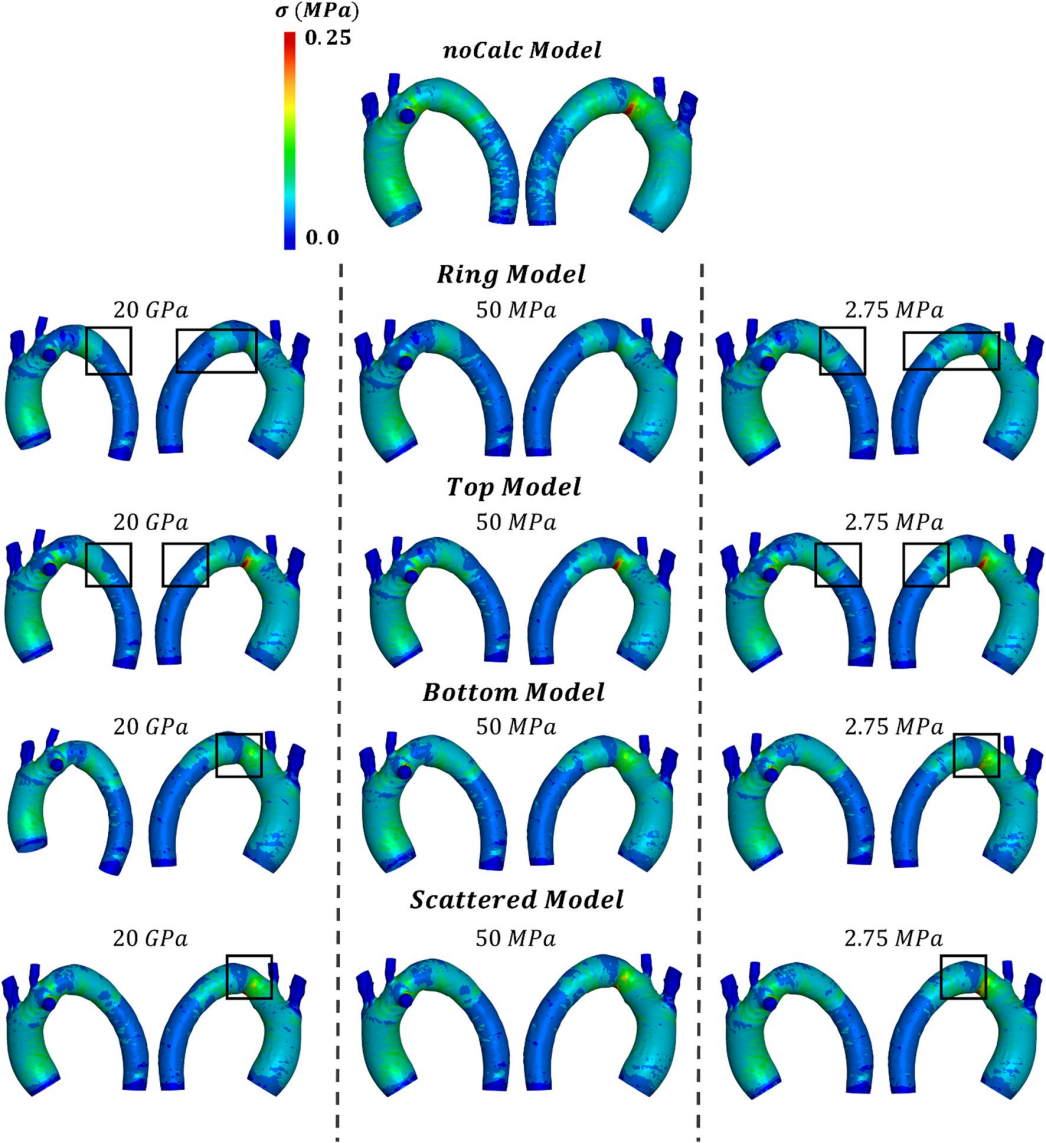
Representation of the von Mises stresses on all the models analyzed. The black squares highlight the region where the calcifications are present and show lower von Mises stresses values

Von Mises stresses on aorta, calcification and on the stent was analyzed on the Ca and noCa models. In the noCa models, the von Mises stresses post-TEVAR procedure were localized mainly in the bottom part of the arch (Fig. 6).

The same higher stresses were observed in the TM where the calcifications were not present in the arch.

For the remaining three models, this pattern was not observed, but the arch was covered by the calcifications that seemed to provide a protective effect on the aortic wall.

In the Ca models having a Young’s modulus of 20 GPa, the stresses on the aorta dropped almost to zero near the calcified regions. However, as the Young modulus of the calcifications was reduced, the stress on the aorta increased. The black rectangle in Fig. 6 showed the region where this pattern can be observed.

**Fig. 7:**
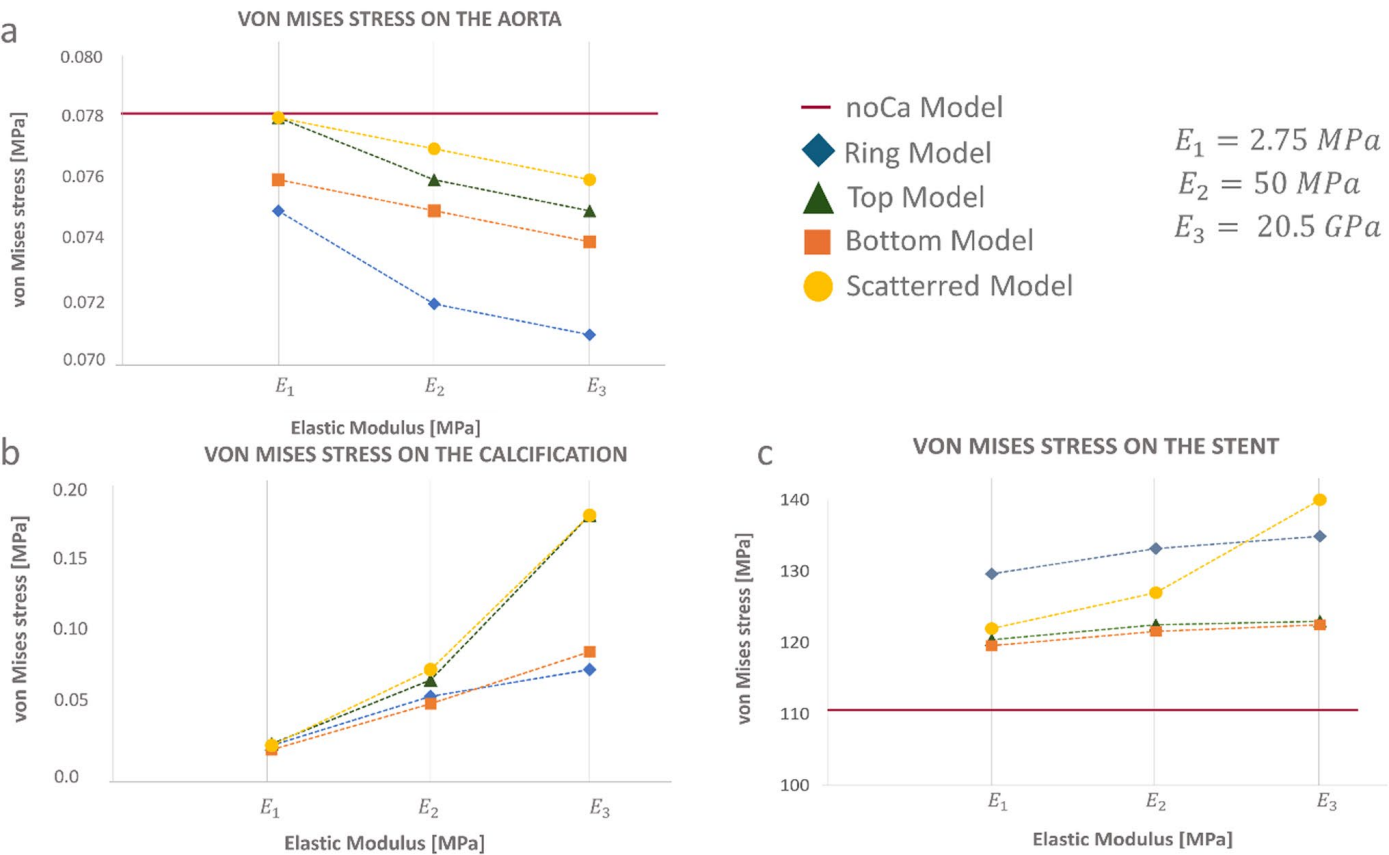
Quantification of the von Mises stresses **a** on the aorta; **b** on the calcifications; **c** on the stent

When the Young modulus reached 2.75 MPa, higher stresses were observed in the arch, similar to the noCa model. This trend was evident not only in the TM but also in the other three Ca models, although with lower stress values. The qualitative analysis was confirmed by quantification of the mean von Mises stress presented in Fig. 7a.

When quantifying the average stresses, with a Young modulus of 2.75 MPa assigned to the calcifications, the stresses on the aorta in the SM and TM matched those observed in the noCa models. The BM and TM, instead, exhibited lower stresses, suggesting that the calcifications seemed to continuously provide a protective effect on the aorta. The model which had the lowest aorta stresses was the RM, while the highest one belonged to the SM.

**Fig. 8:**
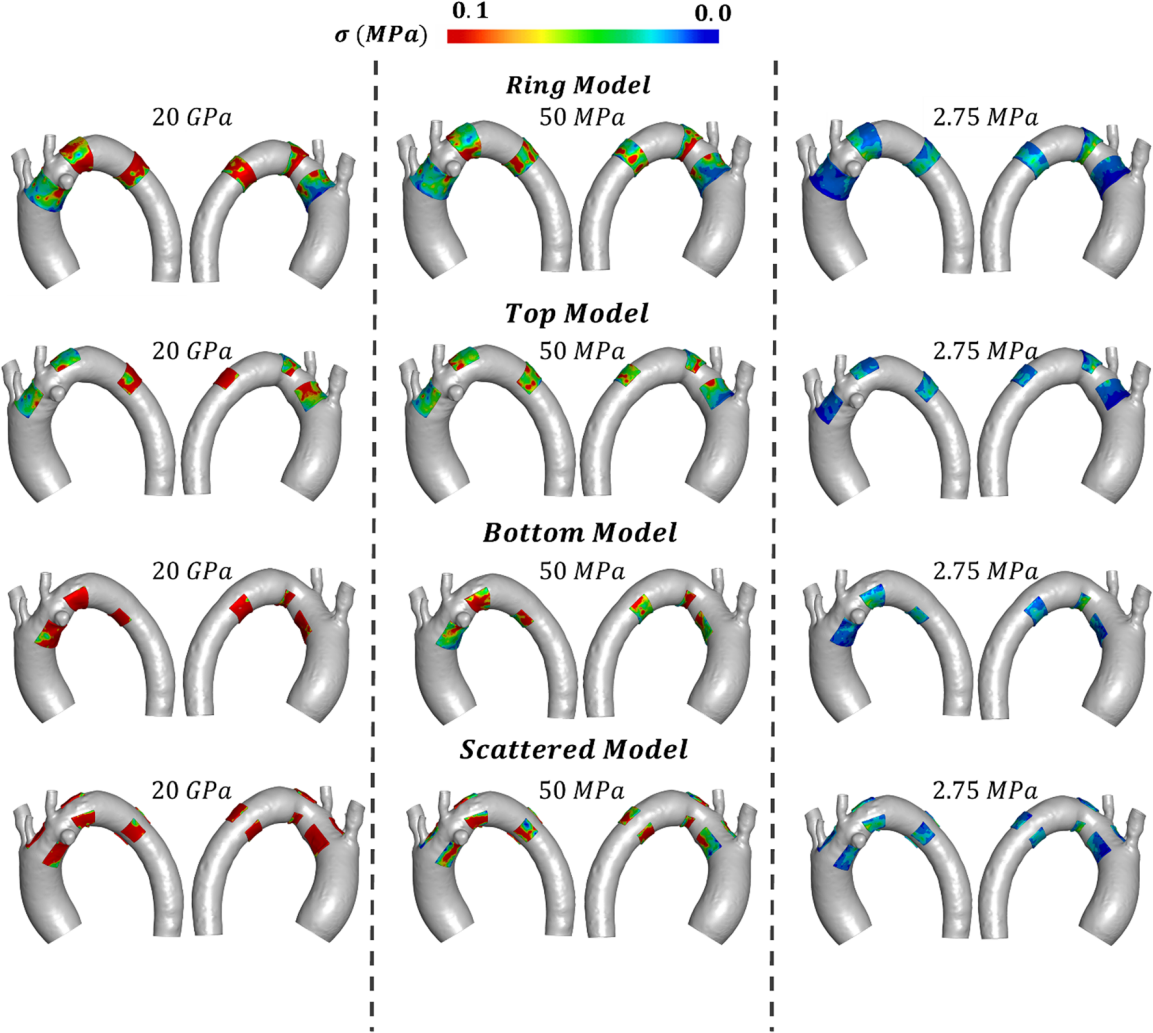
Quantification of the von Mises stresses on the calcification in the four models analyzed

Observing the calcifications von Mises stresses, Fig. 8 highlighted that as the Young modulus of the calcification was reduced from 20 GPa to 2.75 MPa, the stresses on the calcification post-TEVAR implant were highly reduced. Furthermore, the model with the highest von Mises stresses appeared to be in the scattered model, when a 20 GPa Young modulus was assigned to the calcifications. From the stresses representation, the calcifications present in the arch region seemed the most solicited, but when the average von Mises stress values are calculated, the TM was the one that exhibited the highest stresses (Fig. 7b). TM and SM showed the same trend, with a sharp increase in the von Mises stress of the calcification when the Young modulus changed from 50 MPa to 20 GPa. On the other hand, BM and RM showed a more linear behavior as the Young modulus increased from 2.75 MPa to 20 GPa.

To conclude, Fig. 7c shows how stresses on the stent, for the Ca models resulted higher compared to the noCa model. The highest value of the stresses of the stent was observed in the SM in correspondence of 20 GPa calcifications (Fig. 7c). BM and TM, instead, shared the same trend, with average stress values lower than the one calculated on the RM.

### Impact on the contact pressure

**Fig. 9:**
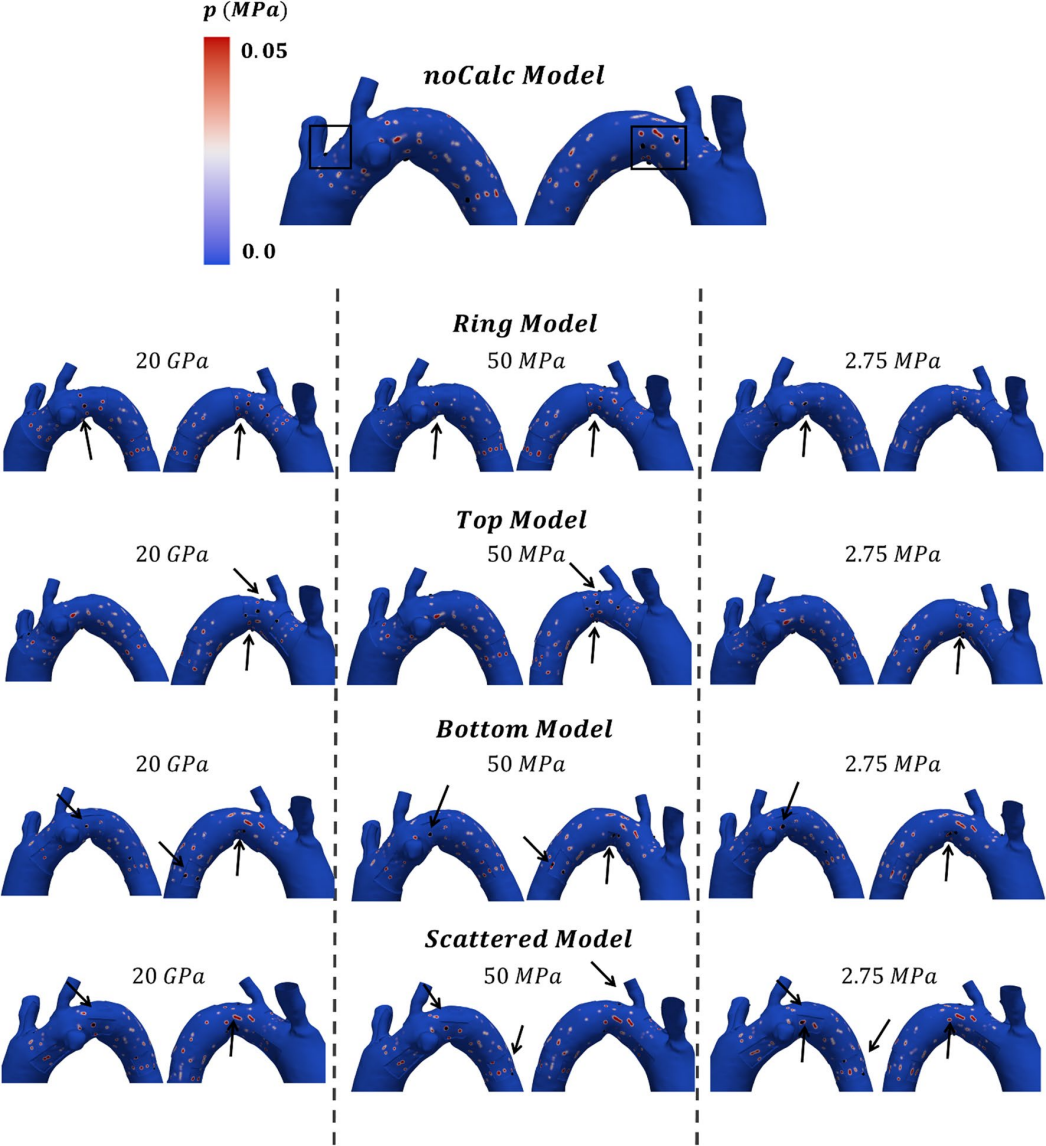
Contact pressure illustration for the different models. The black arrows highlight the region with the highest contact pressures for all the models

**Fig. 10:**
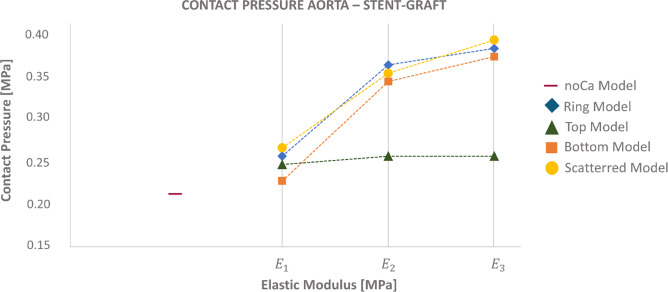
Contact pressure quantification for the Ca and noCa models

Contact pressures were represented on the aorta, with the maximum points highlighted with black arrows (Fig. 9). Almost all the models presented maximum contact pressure values in the arch and in correspondence of the calcifications. All the models showed higher contact pressures as the Young modulus of the calcification increased. The quantification of contact pressure values revealed that the BM exhibited the lowest contact pressures among all models. The values were close to the one observed in the noCa model (Fig. 10).

In contrast, the Ca models showed the same trend with contact pressure values that rapidly increase when the Young modulus assigned to the calcification goes from 2.75 MPa to 50 MPa. A more gradual increase was then observed when the Young modulus goes from 50 MPa to 20 GPa (Fig. 10).

## Discussion

Performing patient-specific FE simulation of TEVAR procedure is important to understand the stent graft behavior in patient-specific anatomies. Ramella et al. in 2022 (Ramella et al. 2022), developed and validated the “tracking method” which consisted in the high-fidelity reproduction of the clinical procedure, by replicating device crimping, tracking and release in the correct aorta landing zone. This procedure was first validated into an idealized aorta model (Ramella et al. 2022) and after into patient-specific anatomies (Ramella et al. 2022; 2023a, b). Their model, however, did not include calcifications that may affect the stent graft deployment in the aorta.

To the best of the authors’ knowledge, this is the first article to investigate the impact of calcification after TEVAR implant, considering explicitly calcifications, which means that aorta and calcifications were modeled as separate parts. McLennan et al in 2023 (McLennan et al. 2023), indeed, observed the impact of calcifications on the aortic stress during EVAR procedure, but reproducing the calcification implicitly, which means by assigning the material properties of the calcification in the correspondent part of the aorta, while other studies focused on preoperative analysis evaluating the stresses on the aortic wall, as already stated in Introduction (Li et al. 2008; Huang et al. 2001).

To validate the patient-specific stent implantation model chosen for this study, our first step was to compare the simulation with the CT of the patient after the stent implantation. The simulation was in very good agreement with the CT-post, with opening area errors lower than 7% for all the stent struts. To be able to understand the quality of the results achieved in this study, it is important to compare them with findings from previous research. Perrin et al. (2015) reached errors in the opening area (OA) comparison up to 35% and Kan et al. (2021) reported errors from 10 to 25%, while Ramella et al. (2022; 2023a, b), found OA percentage errors below 10%. Also in this study, as errors were lower if compared to the other literature studies just described, assessing good reliability and robustness of the results.

This initial step was essential to assess the credibility of our simulations. By confirming that the patient-specific model aligned with the postoperative CT scan, the same model—with the addition of calcifications—could be used to evaluate their impact on the TEVAR simulation. This approach ensured that the effect of the calcifications was properly assessed, minimizing other uncertainties that could affect the results.

In the literature, there are high discrepancies on the quantification of the material properties of the calcifications. To address this variability, we selected three representative values corresponding to the lowest, highest and an intermediate modulus reported by Loore, Marra and Meier, respectively, as outlined earlier (Loree et al. 1994; Marra et al. 2006; Maier et al. 2010). By testing different material properties and placing calcifications in critical regions, this study provides insights into how calcifications affect TEVAR outcomes.

From a qualitative perspective, the simulations showed a good stent graft apposition for all the Ca models when compared with the noCa model. The device was displaced correctly inside the anatomy; nevertheless, the stent overlapping between Ca and noCa model showed a rotation among the stent that could be associated with the presence of the calcifications in the arch.

Evaluating quantitatively the models, a reduction of the opening area was experienced due to the penetration of the calcification in the internal lumen. This observation was associated with Young modulus variations. The calcifications indeed were more compliant as their Young modulus was reduced, allowing the stent to displace more the aorta upon release.

Furthermore, some noteworthy findings were observed when evaluating the von Mises stresses. The calcifications were likely to absorb the stresses which were then reduced on the aorta. This could be observed mainly in the TM which presented a high stress level in the arch, which was observed also in the noCa model, but not in the remaining Ca model where the calcification covered the arch.

Calcifications appeared to function as load-bearing structures by absorbing stresses, thereby reducing the stress on the aorta. However, as the Young’s modulus of the calcifications decreased, this effect diminished. Softer, more compliant calcifications were likely less capable of absorbing stress effectively. Furthermore, RM that had the highest CI experienced the highest decrease in the average von Mises stress, suggesting that the extension of the plaque had an influence on the stress on the aorta.

A load-bearing behavior and the influence of the CI were also observed by Meier et al. (2010); however, direct comparisons were challenging as their analysis focused on calcifications embedded within a thrombus and conducted prior to the EVAR procedure.

This finding was significant, as an increase in wall stress on the aorta was associated with a higher risk of aneurysm rupture (Maier et al. 2010; Farotto et al., 2017). The aneurysm rupture, indeed, happened when the stress on the aortic wall during the cardiac cycle exceeded the strength of the wall (Huang et al. 2001). In this case, however, calcifications helped reducing the average von Mises stresses on the aorta, potentially lowering the likelihood of aneurysm rupture.

On the other hand, the von Mises stresses on the calcifications acted oppositely with respect to the stresses on the aorta. The von Mises stress was higher in the calcified model adopting 20 GPa Young modulus and reduces as the Young modulus was reduced. Unexpectedly, the calcifications with the highest stresses were not the one belonging to the RM, but to the SM and TM. The hypothesis below this result was that in the RM the stresses might have been homogeneously distributed since they cover the entire circumference of the aorta, resulting in an average value lower than the other models.

While von Mises stresses on the aorta and calcifications have been analyzed in previous studies (Huang et al. 2001; Barrett et al. 2018; Luraghi et al. 2019; Ramella et al. 2023b), this work was the first to evaluate the stresses on the stent and the contact pressures between the stent and the aorta. These parameters could give a new perspective on the importance of the impact of calcifications in the TEVAR procedure and the necessity to take them into account when performing in silico studies,

Regarding the stresses on the stent, the Ca models exhibited higher stresses compared to the noCa model. This was because, as the stent was deployed, it encountered stiffer regions in the Ca models, requiring greater forces to fully expand and achieve proper placement.

However, the highest average von Mises stresses were found for the SM with the highest Young modulus of the calcifications (20 GPa). While it was initially expected that the RM, with its constraining geometry, would produce the highest stresses, the SM’s elevated values might be attributed to the specific positioning of the calcifications and the patient-specific geometry. In this case, the alternating presence of aorta and calcifications likely required higher forces for the stent to achieve correct placement.

For what concerned the contact pressure results, the RM showed very high contact pressure: The stent here was constrained in all directions and this could result in high contact pressure values. Higher forces needed to be exerted by the stent graft on the calcifications, particularly when the Young modulus of 20 GPa was adopted, in order to reach its final configuration.

The BM, on the other hand, appeared to have the lowest contact pressures, comparable to the noCa model. This might have been attributed to the fact that in the BM the calcification was positioned in the arch, but the stent encountered a more compliant wall on the opposite side, which might have helped reducing the contact pressure on the model. On the other hand, in the TM there was a calcification in the top part of the arch and the arch itself on the opposite side, which was the most solicited region. This might explain why the TM resulted with contact pressure closer to the RM even if the CI is almost equal to the BM. The SC, similar to the RM and TM, also showed high contact pressures. This was likely influenced by the patient-specific geometry and the distribution of calcifications.

Predicting von Mises stresses and contact pressures on aortas with spotted calcifications (SM) was challenging, as the calcifications could create localized areas where stresses or pressures accumulated due to the patient-specific anatomy. Nevertheless, this model was the closest approximation to a patient-specific aorta with calcifications distributed throughout. Eventually, we accepted these results, but it was important to note that they might not be generalized to all cases.

An exhaustive analysis of the impact of the calcification on the TEVAR procedure was performed considering not only the von Mises stresses values on aorta and calcification, but also the one on the stent, the contact pressure and the opening area comparison. In our view, this comprehensive approach provided a complete understanding of how calcifications influence the mechanical performance of the procedure.

This study, however, has some limitations. The calcification and the aortic material were set as linear elastic, but more suitable formulation could be used. Calcifications indeed were mainly modeled as hyperelastic, homogeneous, incompressible and isotropic material following the Mooney–Rivlin formulation (Huang et al. 2001; Li et al. 2008; Maier et al. 2010; McLennan et al. 2023); also, the aorta was usually modeled with hyperelastic isotropic material following the Rvagan and Vorp formulation (Maier et al. 2010; Ramella et al. 2024). Nonetheless, the validation in a patient-specific case without calcification showed very good agreement between simulation and segmentation. Moreover, since all models were assumed to be linear elastic, the overall comparison remains acceptable.

In addition, the thickness of the calcification was set as constant, but it might have a non-negligible impact on the TEVAR procedure. Moreover, an idealized model of calcification was developed, and a patient-specific anatomy was chosen. While this approach provided initial insights, it might limit the generalizability of the findings. Future studies should include a broader range of patient-specific geometries that inherently account for calcifications to improve the robustness of the conclusions.

Lastly, explicitly modeling calcifications revealed stress concentrations within the deposits, suggesting the need for appropriate failure criteria for calcified regions. The rupture of the calcified plaque can indeed cause more complications, leading to a worsening of the patient condition.

Further research is also needed from a fluid dynamic point of view for better understanding risks of migration and endoleaks.

## Conclusion

This study showed that the calcifications appeared to function as load-bearing structures, reducing stress on the aorta while increasing stress on the stent graft and on the calcifications themselves. Additionally, although the opening area was smaller in the Ca models compared to the noCa model, the qualitative comparison of device displacement remained unchanged. Furthermore, a Young’s modulus of 2.75 MPa seemed to be very similar to the properties of aortic tissue. This was reflected in the results, as all evaluated parameters showed values comparable to those of the noCa models. This suggests that a Young’s modulus of 2.75 MPa may not be suitable for accurately representing calcifications and could be excluded from consideration in future calcification models.

Calcifications in the landing zones are considered as an issue when TEVAR management is performed.

Our analysis regarding stresses and device placement can be a good starting point on effectively analyzing more in depth and with patient-specific anatomies, the effect of calcification on TEVAR outcomes. This might improve procedural planning and device selection in the clinical procedure.

## Supplementary Information

Below is the link to the electronic supplementary material.

Supplementary file1 (DOCX 397 kb)

## Data Availability

No datasets were generated or analysed during the current study.

## References

[CR1] Aldieri A, Curreli C, Szyszko JA et al (2023) Credibility assessment of computational models according to ASME V&V40: application to the Bologna biomechanical computed tomography solution. Comput Methods Programs Biomed 240:107727. 10.1016/j.cmpb.2023.107727 37523955 10.1016/j.cmpb.2023.107727

[CR2] Barrett HE, Cunnane EM, Hidayat H et al (2018) On the influence of wall calcification and intraluminal thrombus on prediction of abdominal aortic aneurysm rupture. J Vasc Surg 67:1234-1246.e2. 10.1016/j.jvs.2017.05.086 28899569 10.1016/j.jvs.2017.05.086

[CR3] Desai MY, Cremer PC, Schoenhagen P (2018) Thoracic aortic calcification: diagnostic, prognostic, and management considerations. JACC Cardiovasc Imaging 11:1012–1026. 10.1016/j.jcmg.2018.03.023 29976300 10.1016/j.jcmg.2018.03.023

[CR32] Farotto D, Segers P, Meuris B, Vander Sloten J, Famaey N (2018) The role of biomechanics in aortic aneurysm management: requirements open problems and future prospects. J Mech Behav Biomed Mater 77295-307. 10.1016/j.jmbbm.2017.08.019 28961516 10.1016/j.jmbbm.2017.08.019

[CR4] Findeiss LK, Cody ME (2011) Endovascular repair of thoracic aortic aneurysms. Semin Intervent Radiol 28:107–117. 10.1055/s-0031-1273945 22379281 10.1055/s-0031-1273945 PMC3140248

[CR29] Grabenwöger M, Alfonso F, Bachet J, Bonser R, Czerny M, Eggebrecht H, Evangelista A, Fattori R, Jakob H, Lönn L, Nienaber CA, Rocchi H, Rousseau M, Thompson E, Weigang R, Erbel (2012) Thoracic Endovascular Aortic Repair (TEVAR) for the treatment of aortic diseases: a position statement from the European Association for Cardio-Thoracic Surgery (EACTS) and the European Society of Cardiology (ESC) in collaboration with the European Association of Percutaneous Cardiovascular Interventions (EAPCI). Eur Heart J 33(13):1558–1563. 10.1093/eurheartj/ehs074 10.1093/eurheartj/ehs074 22561257

[CR5] Gui Z, Shao C, Zhan Y et al (2024) Vascular calcification: high incidence sites, distribution, and detection. Cardiovasc Pathol. 10.1016/j.carpath.2024.107667 10.1016/j.carpath.2024.107667 38866090

[CR6] Huang H, Virmani R, Younis H et al (2001) The impact of calcification on the biomechanical stability of atherosclerotic plaques. Circulation 103:1051–1056. 10.1161/01.CIR.103.8.1051 11222465 10.1161/01.cir.103.8.1051

[CR7] Kan X, Ma T, Lin J et al (2021) Patient - specific simulation of stent—graft deployment in type B aortic dissection : model development and validation. Biomech Model Mechanobiol 20:2247–2258. 10.1007/s10237-021-01504-x 34431034 10.1007/s10237-021-01504-x PMC8595232

[CR30] Ladich E, Yahagi K, Romero ME, Virmani R (2016) Vascular diseases: aortitis aortic aneurysms and vascular calcification. Cardiovasc Pathol 25(5):432–441. 10.1016/j.carpath.2016.07.002 10.1016/j.carpath.2016.07.002 27526100

[CR8] Le Bars F, Charbonneau E, Leurent G, Kaladji A (2022) First report of endovascular treatment of symptomatic coral reef aorta in the aortic arch. JTCVS Tech 12:17–20. 10.1016/j.xjtc.2022.01.010 35403050 10.1016/j.xjtc.2022.01.010 PMC8987378

[CR9] Li ZY, U-King-Im J, Tang TY et al (2008) Impact of calcification and intraluminal thrombus on the computed wall stresses of abdominal aortic aneurysm. J Vasc Surg 47:928–935. 10.1016/j.jvs.2008.01.006 18372154 10.1016/j.jvs.2008.01.006

[CR10] Loree HM, Grodzinsky AJ, Park SY et al (1994) Static circumferential tangential modulus of human atherosclerotic tissue. J Biomech 27:195–204. 10.1016/0021-9290(94)90209-7 8132688 10.1016/0021-9290(94)90209-7

[CR11] Luraghi G, Migliavacca F, García-González A et al (2019) On the modeling of patient-specific transcatheter aortic valve replacement: a fluid-structure interaction approach. Cardiovasc Eng Technol 10:437–455. 10.1007/s13239-019-00427-0 31309527 10.1007/s13239-019-00427-0

[CR12] Maier A, Gee MW, Reeps C et al (2010) Impact of calcifications on patient-specific wall stress analysis of abdominal aortic aneurysms. Biomech Model Mechanobiol 9:511–521. 10.1007/s10237-010-0191-0 20143120 10.1007/s10237-010-0191-0

[CR13] Marra SP, Daghlian CP, Fillinger MF, Kennedy FE (2006) Elemental composition, morphology and mechanical properties of calcified deposits obtained from abdominal aortic aneurysms. Acta Biomater 2:515–520. 10.1016/j.actbio.2006.05.003 16839827 10.1016/j.actbio.2006.05.003

[CR14] McLennan S, Soulez G, Mongrain R et al (2022) Impact of calcification modeling to improve image fusion accuracy for endovascular aortic aneurysm repair. Int J Numer Method Biomed Eng 38:1–9. 10.1002/cnm.3556 10.1002/cnm.3556 34854247

[CR15] McLennan S, Soulez G, Mohammadi H et al (2023) A patient-specific numerical model to assess the impact of calcification stress during endovascular aortic aneurysm repair. Procedia Struct Integrity 49:51–58. 10.1016/j.prostr.2023.10.009

[CR16] Nation DA, Wang GJ (2015) TEVAR: endovascular repair of the thoracic aorta. Semin Intervent Radiol 32:265–271. 10.1055/s-0035-1558824 26327745 10.1055/s-0035-1558824 PMC4540616

[CR17] O’Leary SA, Mulvihill JJ, Barrett HE et al (2015) Determining the influence of calcification on the failure properties of abdominal aortic aneurysm (AAA) tissue. J Mech Behav Biomed Mater 42:154–167. 10.1016/j.jmbbm.2014.11.005 25482218 10.1016/j.jmbbm.2014.11.005

[CR18] Perrin D, Badel P, Orgéas L et al (2015) Patient-specific numerical simulation of stent-graft deployment: validation on three clinical cases. J Biomech 48:1868–1875. 10.1016/j.jbiomech.2015.04.031 25979382 10.1016/j.jbiomech.2015.04.031

[CR19] Ramella A, Migliavacca F, Rodriguez Matas JF et al (2022) Validation and verification of high-fidelity simulations of thoracic stent-graft implantation. Ann Biomed Eng 50:1941–1953. 10.1007/s10439-022-03014-y 35854187 10.1007/s10439-022-03014-y PMC9794542

[CR20] Ramella A, Migliavacca F, Rodriguez Matas JF et al (2023b) Applicability assessment for in-silico patient-specific TEVAR procedures. J Biomech 146:111423. 10.1016/j.jbiomech.2022.111423 36584506 10.1016/j.jbiomech.2022.111423

[CR21] Ramella A, Lissoni V, Bridio S et al (2024) On the necessity to include arterial pre-stress in patient-specific simulations of minimally invasive procedures. Biomech Model Mechanobiol 23:525–537. 10.1007/s10237-023-01789-0 38063955 10.1007/s10237-023-01789-0 PMC10963513

[CR22] Ramella A, Migliavacca F, Rodriguez Matas JF (2023a) A numerical finite element methodology of the EVAR procedure. In: Convegno Nazionale di Bioingegneria, p 21–24

[CR31] Reeps C, Maier A, Pelisek J, Härtl F, Grabher-Meier V, Wall WA, Essler M, Eckstein HH, Gee MW (2013) Measuring and modeling patient-specific distributions of material properties in abdominal aortic aneurysm wall. Biomech Model Mechanobiol 12(4):717–733. 10.1007/s10237-012-0436-1 10.1007/s10237-012-0436-1 22955570

[CR23] Speelman L, Bohra A, Bosboom EMH et al (2007) Effects of wall calcifications in patient-specific wall stress analyses of abdominal aortic aneurysms. J Biomech Eng 129:105–109. 10.1115/1.2401189 17227104 10.1115/1.2401189

[CR24] Takayama Y, Yasuda Y, Suzuki S et al (2016) Relationship between abdominal aortic and coronary artery calcification as detected by computed tomography in chronic kidney disease patients. Heart Vessel 31:1030–1037. 10.1007/s00380-015-0712-y 10.1007/s00380-015-0712-y 26164596

[CR25] Upchurch GR, Escobar GA, Azizzadeh A et al (2021) Society for vascular surgery clinical practice guidelines of thoracic endovascular aortic repair for descending thoracic aortic aneurysms. J Vasc Surg 73:55S-83S. 10.1016/j.jvs.2020.05.076 32628988 10.1016/j.jvs.2020.05.076

[CR26] Van Bogerijen GHW, Tolenaar JL, Conti M et al (2013) Contemporary role of computational analysis in endovascular treatment for thoracic aortic disease. AORTA 1:171–181. 10.12945/j.aorta.2013.13-003 26798690 10.12945/j.aorta.2013.13-003 PMC4682739

[CR27] Vos A, Houben IB, Celeng C et al (2023) Aortic calcification: a postmortem CT validation study in a middle-aged population. Eur J Radiol 159:110687. 10.1016/j.ejrad.2023.110687 36610325 10.1016/j.ejrad.2023.110687

[CR28] Wong KKL, Thavornpattanapong P, Cheung SCP et al (2012) Effect of calcification on the mechanical stability of plaque based on a three-dimensional carotid bifurcation model. BMC Cardiovasc Disord. 10.1186/1471-2261-12-7 10.1186/1471-2261-12-7 PMC3310807 22336469

