# Supplementary material for: Assessing calcification effects in TEVAR procedures: a computational analysis
Source: Biomech Model Mechanobiol. 2025 Sep 12;24(6):1913–26. doi: 10.1007/s10237-025-01998-9 (PMC12618359; doi:10.1007/s10237-025-01998-9)
Supplement: Supplementary file 1 — Supplementary file1 (DOCX 397 kb) [file 10237_2025_1998_MOESM1_ESM.docx]

**Appendix:**

Detailed Table on the material properties of calcification, aorta and stent-graft used in the paper.

| **Component** | **Material Type** | **Material properties** | **Literature reference** |
| --- | --- | --- | --- |
| Calcification | Linear Elastic | E=2.75MPa  E=50MPa  E=20GPa  ν=0.4  ρ=0.12g/mm^3 | (Loree et al. 1994; Marra et al. 2006; Maier et al. 2010; McLennan et al. 2022) |
| Aorta | Linear Elastic | 2 MPa  ν=0.49  ρ=0.00112 g/mm^3 | (Ramella et al. 2024) |
| Stent | Shape Memory Alloy (*) | E_austenite_ = 57,5 MPa ν_austenite_ = 0.3  E_martensite_ = 47,8MPa  ν_martensite_ = 0.3  ε=0.063  σ^S^_L_=550MPa  σ^E^_L_=620MPa  σ^S^_U_=450MPa  σ^E^_U_=250MPa  α= 0.0279 | (Ramella et al. 2022) |
| Graft | Linear Isotropic Elastic Fabric, no resistance to compression | 1080 MPa  ν= 0.35 | (Ramella et al. 2022) |

Tabella 1 – Detailed description of the material properties assigned to: Calcification, aorta, stent and graft ( E= Young Modulus, ν= Poisson ratio, ρ= density)

* Parameters used to model Nitinol are: Austenite (E_austenite_); elastic modulus of Martensite (E_martensite_); Austenite and Martensite Poisson’s ratio (ν); elastic modulus of starting value for the forward phase transformation (conversion of austenite into martensite) (σ^S^_L_); final value for the forward phase transformation (σ^E^_L_); starting value for the reverse phase transformation (conversion of martensite into austenite) (σ^S^_U_); final value for the reverse phase transformation (σ^E^_U_); maximum residual strain (ε) and parameter measuring the difference between material responses in tension and compression (α) (Ramella et al. 2022).

Detailed description of the steps of the TEVAR simulation:

The TEVAR simulation performed in this article follow the “tracking method” (Figure 1) of Ramella et al. (Ramella et al. 2022), which consist of three steps:

1. Crimping phase: the stent-graft is crimped using a cylinder. Soft penalty-based contact with a friction coefficient of 0.1 is used to crimp the device inside a catheter of 10 mm in diameter.
2. Tracking phase: the device is displaced along the centerline of the aorta until the correct landing zone is reached. The stent-graft is in contact with the catheter during the displacement phase to remain crimped. Soft penalty-based contact with null friction coefficient is used between device and catheter.
3. Release phase: The device is gradually released inside the aorta by progressively deactivating the contact constraints between the device and catheter, while a soft penalty-based contact between aorta/calcifications and the device is established, using a friction coefficient of 0.5.


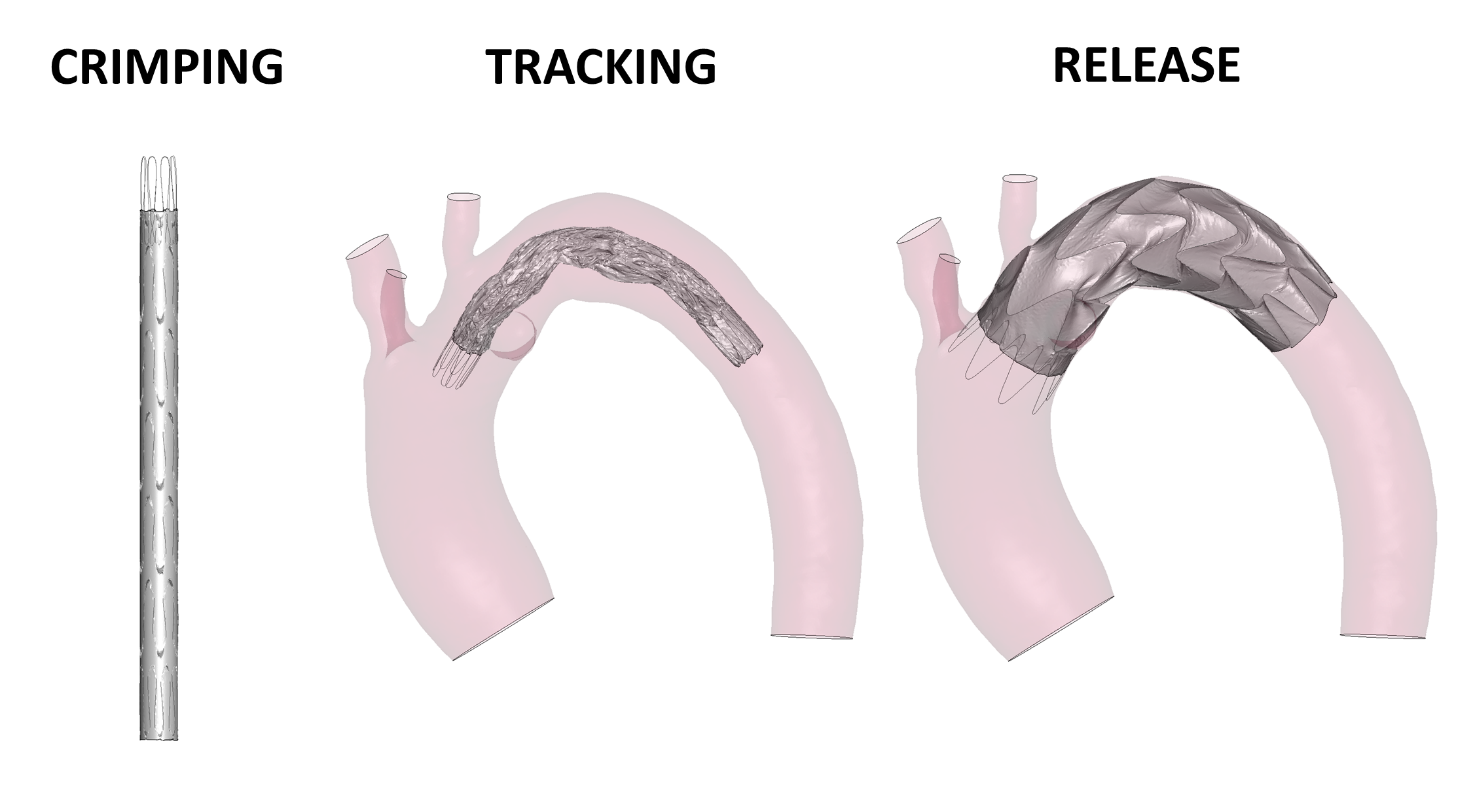


Figure **1-** The figure shows the three steps of the TEVAR procedure following the 'tracking method' (Ramella et al. 2022).
